# Second Dose of Epinephrine for Anaphylaxis in the First Aid Setting: A Scoping Review

**DOI:** 10.7759/cureus.11401

**Published:** 2020-11-09

**Authors:** Jestin N Carlson, Scott Cook, Therese Djarv, Jeff A Woodin, Eunice Singletary, David A Zideman

**Affiliations:** 1 Emergency, Saint Vincent Hospital, Erie, USA; 2 Emergency, Emergency Services of Montgomery, Montgomery, USA; 3 Emergency Medicine, Karolinska Institute, Stockholm, SWE; 4 Emergency, Tualatin Valley Fire & Rescue, Tigard, USA; 5 Emergency Medicine, University of Virginia, Charlottesville, USA; 6 Pre-Hospital Emergency Medicine, Thames Valley Air Ambulance, Oxford, GBR

**Keywords:** anaphylaxis, first-aid, epinephrine

## Abstract

Anaphylaxis is a life-threatening hypersensitivity reaction where rapid, early administration of epinephrine (adrenaline) can be lifesaving in the first aid setting. There are instances where a single dose of epinephrine does not relieve symptoms and a second dose may be required to further mitigate symptoms and preserve life.

We performed a scoping review as part of an update to a previously conducted International Liaison Committee on Resuscitation First Aid Task Force (ILCOR) review. PubMed and Embase were searched using the strategy from the 2015 ILCOR review (dates January 1, 2015 to October 22, 2019) and a review of the grey literature (all dates up to November 18, 2019) was performed to identify data on the requirement, use, and effectiveness of a second dose of epinephrine. Each search was rerun on June 26, 2020. We included all human studies of adults and children with an English abstract. Critical outcomes included resolution of symptoms, adverse effects, and complications of second dosing of epinephrine in the treatment of acute anaphylaxis. Included studies are presented descriptively.

Our updated search identified 909 potential sources, 890 from the published literature and 19 from the grey literature. After full text review, two studies met our eligibility criteria (Campbell et al. and Akari et al.). For the outcome of resolution of symptoms, both studies found that two or more doses of epinephrine were required in 8% of 582 patients and 28% of 18 patients, respectively, with anaphylaxis requiring treatment with epinephrine. The other a priori outcomes were not reported.

This scoping review identified limited evidence regarding the use of a second dose of epinephrine for anaphylaxis in the first aid setting, however, due to the potential benefit, it is reasonable to administer a second dose when symptoms of severe anaphylaxis fail to resolve following an initial dose. Given the potential mortality associated with anaphylaxis, further research is needed to better identify individuals who may benefit from a second dose of epinephrine.

## Introduction and background

Anaphylaxis is a life-threatening hypersensitivity condition that can be fatal if it is not quickly recognized and treated. Causes of anaphylactic reactions include food, medicines, insect sting/venom, general anesthetics, contrast agents and latex, with medication being the leading cause of anaphylaxis in the United States [1]. Although delayed presentation can rarely occur, fatal anaphylaxis can occur rapidly after exposure - five minutes for an iatrogenic reaction; 15 minutes for an insect sting/venom reaction; and 30 minutes for food reactions, highlighting the need for strategies to manage anaphylaxis in the first aid setting [2].

Intramuscular injection of epinephrine is the primary rescue drug recommended for use in the acute treatment of anaphylaxis. The World Allergy Organization recommends the administration of epinephrine (adrenaline) by intramuscular injection into the mid-lateral thigh, 0.01 mg/kg of a 1:1000 (1 mg/mL) solution to a maximum of 0.5 mg in adults and 0.3 mg in children [3]. These injections are usually administered by autoinjector syringes and can be self-administered or given by family, friends or a first aid provider, providing suitable training has been given. There is no absolute contraindication for epinephrine administration during anaphylaxis and epinephrine should be administered as soon as possible if anaphylaxis is suspected [4-7]. Some individuals may not respond to a single dose of epinephrine and benefit from additional doses of epinephrine in addition to other medications (e.g., histamine blocking medications) [4, 6]. The utility of a second dose of intramuscular epinephrine in anaphylaxis in the first aid setting is unknown; and while there is theoretical benefit, the potential harms, such as myocardial infarction, are also unknown. We performed a scoping review to understand the current state of the literature regarding the utility of a second dose of epinephrine in anaphylaxis. Among adults and children experiencing severe anaphylaxis requiring the use of epinephrine (P), does administration of a second dose of epinephrine (I), compared with administration of only one dose (C), change resolution of symptoms, adverse effects, complications (O)?

## Review

This scoping review was performed as part of the ILCOR continuous evidence evaluation process, conducted by the ILCOR First Aid Task Force Scoping Review team for the 2020 Consensus on Science with Treatment Recommendations (CoSTR) [8]. This topic was last reviewed in 2015 and a CoSTR was published and led to recommendations from the American Heart Association (AHA) and American Red Cross (ARC) and European Resuscitation Council (ERC) [4-7].

Scoping search strategy

We performed two structured searches; one of the published literature using PubMed and Embase, and a second, grey literature search using Google.com in an attempt to identify knowledge gaps in this area. For the published literature, we re-ran the existing search strategy as used for the ILCOR CoSTR [4, 6]: from January 1, 2014 to October 22, 2019 to ensure we captured all data published since the 2015 search (Appendix). There were no language restrictions for the literature search as long as there was an English abstract. The grey literature search was run on November 18, 2019 (Appendix; Table 2) with no restrictions on date or language. We repeated the published literature and grey searches on June 26, 2020.

The population included adults and children experiencing anaphylaxis requiring the use of epinephrine. We included all human studies with no restrictions on age. Randomized controlled trials (RCTs) and non-randomized studies (non-randomized controlled trials, uninterrupted time series, controlled before-and-after studies, cohort studies) were eligible for inclusion. Studies not reporting on our selected outcomes and those without an English language abstract were excluded. Critical outcomes for the 2015 review included resolution of symptoms, adverse effects and complications and these were maintained for this review [4, 6]. Given the nature of this scoping review, we also considered outcomes as evaluated in newly included articles; however, these were not identified or listed by order of importance.

For the published literature search, three independent reviewers (JNC, TD and JAW) screened the title and abstract of each article. Two independent reviewers (JNC and JAW) then performed a full text review of potential articles to determine the final included articles. For the grey literature search, one reviewer (JNC) performed the initial search and identified potential sources. Two independent reviewers (TD and JAW) then reviewed these sources to identify any additional key sources of information. In cases of discrepancy, an additional reviewer (DAZ) adjudicated the discrepancy. We present descriptive summaries of the final included manuscripts.

Results

Previous Search (2015)

The previous 2015 CoSTR search strategy identified 1632 papers of which 10 met inclusion criteria from which nine could be pooled for analysis (Table 1). The systematic review found benefit for the outcome of resolution of symptoms for giving a second or multiple doses of epinephrine to individuals not responding to a first dose (risk ratio [RR], 1.16; 95% confidence interval [CI], 1.13-1.20) but evidence was deemed to be very-low-certainty (downgraded for risk of bias and confounding) [9-17]. In addition, for the critical outcome of resolution of symptoms, very-low-quality evidence (downgraded for risk of bias) from one observational study showed no difference between the percentage of resolved reactions in an ambulance service routinely using two doses of epinephrine compared with an ambulance service using a single dose (RR, 0.97; 95% CI, 0.9-1.04) [18]. Pooled data from nine observational studies identified in the 2015 ILCOR review found resolution of symptoms of anaphylaxis following a single dose of epinephrine in 74%. No evidence was identified addressing outcomes of adverse effects or complications.

**Table 1:** Details of manuscripts identified in the original search from 2015 and the updated scoping review.

Citation	Study Design	Key Findings
From Previous Search (2015)		
Oren et al. (2007) [9]	Type: Retrospective chart review, Age: not specified, Sample: n = 19	16% were treated with >1 dose of epinephrine.
Jarvinen et al. (2008) [10]	Type: Observational study, Age: 6 m-17.5 years, Sample: n = 95	>1 dose was administered in 12 (13%) and 3 doses in an additional 6 (6%).
Korenblat et al. (1999) [11]	Type: Retrospective study, Age: 4 to 76 years, Sample: n = 105	>35% were administered >1 dose of epinephrine.
Banerji et al. (2010) [12]	Type: Observational study, Age: Adults, Sample: n = 1286	17% were given >1 dose of epinephrine.
Rudders et al. (2010) [13]	Type: Retrospective chart review, Age: <18 years old, Sample: n = 605	12% received a second dose of epinephrine.
Rudders et al. (2010) [14]	Type: Retrospective chart review, Age: 14-51 years, Sample: n = 40	16% received a second dose of epinephrine.
Noimark et al. (2012) [15]	Type: Questionnaire, Age: pediatrics, Sample: n = 41	31.7% received >1 dose of epinephrine
Ellis and Brown (2013) [18]	Type: Retrospective study, Age: not specified, Sample: n = 195 service A n = 231 service B	Service A - 6.7% received >1 dose of epinephrine, Service B - 0 received >1 dose of epinephrine
Inoue and Yamamoto (2013) [16]	Type: Observational study, Age: 2 months to 14 years, Sample: n = 35	4.9% received >1 dose of epinephrine
Tsuang et al. (2013) [17]	Type: Observational study, Age: 4 months – 7 years, Sample: n = 166	10% received >1 dose of epinephrine
From Updated Search		
Campbell et al. (2015) [19]	Type: Combined retrospective chart review and prospective observational cohort, Age: all, Sample: n = 582 (n = 532 retrospective; n = 50 prospective)	8% received > 1 dose of epinephrine and this was associated with greater odds of being admitted to the hospital
Araki et al. (2018) [20]	Type: Oral food challenge, Age: 1-14 years, Sample: n = 42	18 patients developed anaphylaxis of which 5 patients (28%) received >1 dose of epinephrine

New Scoping Review (2015 to 2020)

**Figure 1:**
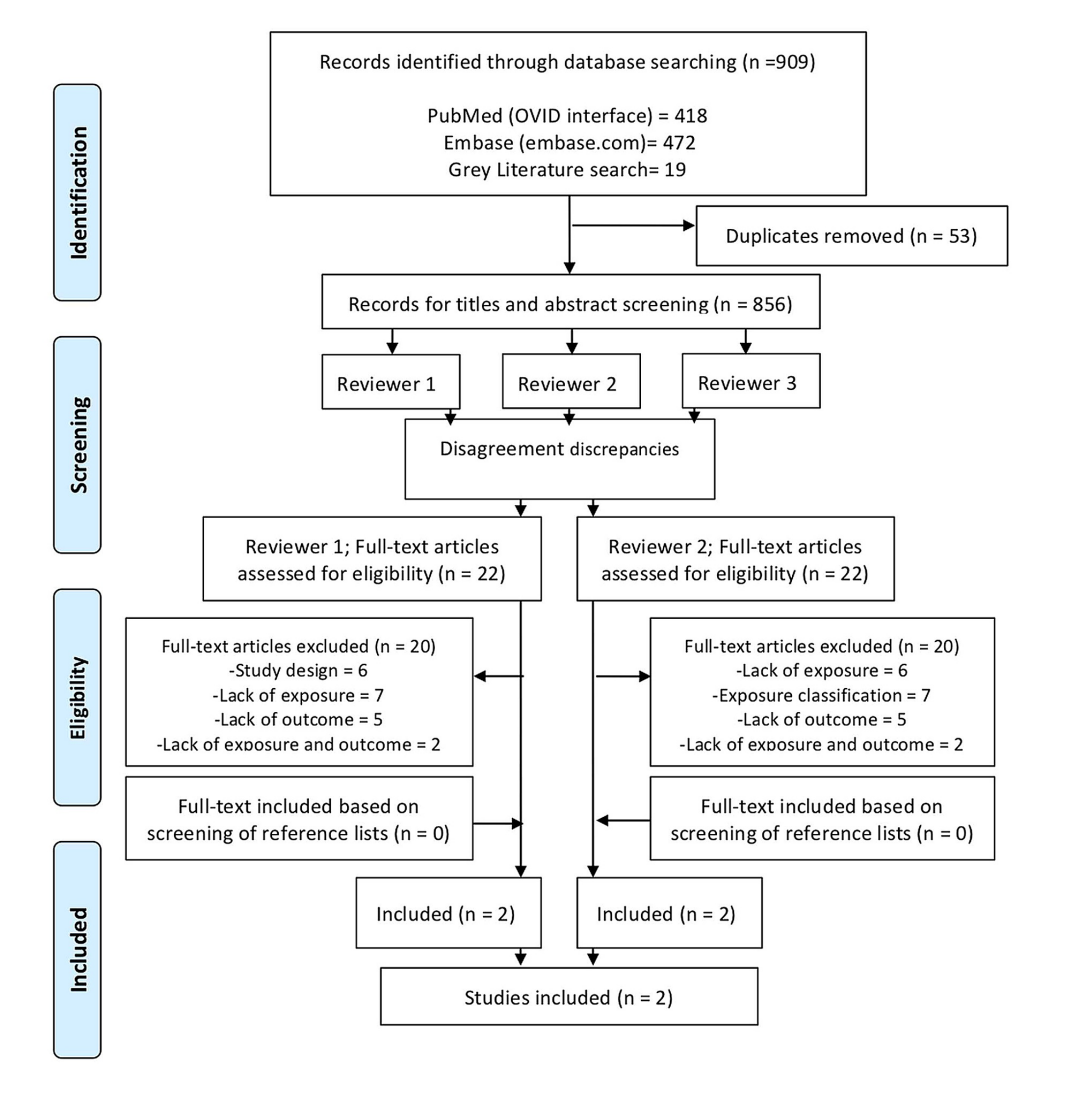
PRISMA diagram of included studies.

Our updated literature search identified 909 potential articles (Figure 1). After removal of duplicates and screening by three reviewers, 22 articles were identified for full text review. Two studies were identified that met final inclusion criteria [19, 20]. These studies compared outcomes between patients who received a single dose of epinephrine and those who received a second dose of epinephrine or multi-dose epinephrine for the treatment of anaphylaxis. Characteristics of included studies from the 2015 CoSTR and the scoping review can be found in Table 1.

Campbell et al. performed a combined retrospective chart review and prospective, observational study of 582 patients who presented to the emergency department (ED) (n = 532 retrospective and 50 prospective) with anaphylaxis of which 45 had multiple doses of epinephrine administered [19]. Receiving more than one dose of epinephrine was associated with higher rates of admission to a general medical floor (odds ratio [OR] 2.8; 95% [CI] 1.1-7.2) or the intensive care unit (OR 7.6; 95% CI 3.7-15.6). In addition, a lower proportion of patients who received at least two doses of epinephrine were discharged home (9/45 [20%] compared with 244/537 [45%]; p = 0.002]. No data were reported on the critical outcomes of resolution of symptoms, adverse effects or complications.

Araki et al. performed an oral food challenge in 42 patients; 18 required epinephrine to treat their anaphylaxis, with five requiring multiple doses [20]. The authors reported that patients with anaphylaxis who received multiple doses of epinephrine presented with higher rates of cardiovascular, neurologic and gastrointestinal symptoms, although we were unable to estimate the exact rates from the published data. During the course of the study, all five patients who received a second dose of epinephrine had resolution of symptoms, although the exact duration and how this compared to those patients not receiving a second dose of epinephrine was unable to be estimated from the published data. No data were reported on the critical outcomes of survival, time to resolution of symptoms or hospital admission.

Our grey literature search identified 19 potential additional sources of information but none of these addressed the outcomes of our PICOST and were not included in this scoping review.

Discussion

Our scoping review identified two additional studies to the 2015 ILCOR review, relevant to the administration of a second dose of epinephrine for the treatment of anaphylaxis but none specific to the first aid setting. Similar to the studies included in a 2015 ILCOR review on the topic, the studies identified in this review were retrospective in nature and suffered from confounding by indication whereby it was unclear why patients received a second dose of epinephrine. As such, interpreting outcomes such as survival and hospital admission may be complicated by the fact that individuals with more severe anaphylaxis may receive additional doses of epinephrine. In these cases, the outcome (e.g., hospital admission) may not be related to the medication administered but instead be a marker of illness severity alone, as also evidenced by the second dose of epinephrine. Neither of the two included studies were performed in the first aid setting. As such, how these findings relate to the first aid setting and the potential need for advanced care (e.g., activation of emergency medical services) if additional epinephrine is needed is unclear.

We utilized the outcomes from the 2015 ILCOR search. Alternative outcomes were identified through this scoping review (hospital admission, time to resolution of symptoms, etc.) that may need to be considered in future reviews. In reviewing manuscripts for this review, we identified one study that sought to determine predictors of repeated doses of epinephrine, such as wheezing, cyanosis or hypotension [21]. While this was outside the scope of this review, this topic is relevant to the field of anaphylaxis management and epinephrine administration and may benefit from a specific scoping or systematic review in the future. We did not identify any prospective, randomized trials comparing the efficacy of a second dose of epinephrine.

Several issues still remain regarding the administration of epinephrine in the first aid setting. First, it is unclear how accurately first aid providers can correctly identify anaphylaxis. If it is identified correctly and epinephrine is administered, there is uncertainty around the initial epinephrine dosing and the need for a second dose. Second, in some countries, the initial dose of epinephrine administered may be less than that recommended in other countries, 0.3 mg intramuscular epinephrine in the USA compared with 0.5 mg in Europe, potentially leading to a greater chance of requiring a second dose. Future work will be needed to evaluate how the size of initial dose of epinephrine impacts the need for repeat dosing in the first aid setting.

There are several limitations to this review. Although this scoping review has not identified sufficient new evidence to prompt a further systematic review following on from the 2015 Systematic Review and CoSTR, it highlights important gaps in research [4-7].

The identified studies suffer from confounding by indication, limiting the understanding of the risks and benefits of a second (or multi-dose) administration of epinephrine in the setting of anaphylaxis. Additional research is needed to evaluate the parameters for a second or multi-dose epinephrine administration in the first aid setting. Only short term, surrogate outcomes were evaluated. Future studies should document the suspected etiology of anaphylaxis, survival, resolution of symptoms, time to resolution of symptoms and need for hospital admission. The ideal initial dose of epinephrine for anaphylaxis is unclear. The dosing of epinephrine autoinjectors varies by geographic region (Adult intramuscular autoinjector dose: 0.3 mg in the United States and 0.5 mg in Europe) and therefore the need for additional doses of epinephrine may vary based on the initial dose delivered.

## Conclusions

This scoping review, conducted as an update to a 2015 ILCOR review of the same topic, found two additional studies evaluating a second dose of epinephrine for anaphylaxis. The limited additional literature identified in this scoping review does not support development of a systematic review, but highlights the need for future research to better understand the role of multi-dose epinephrine in the first aid setting.

## Figures and Tables

**Figure 2:**
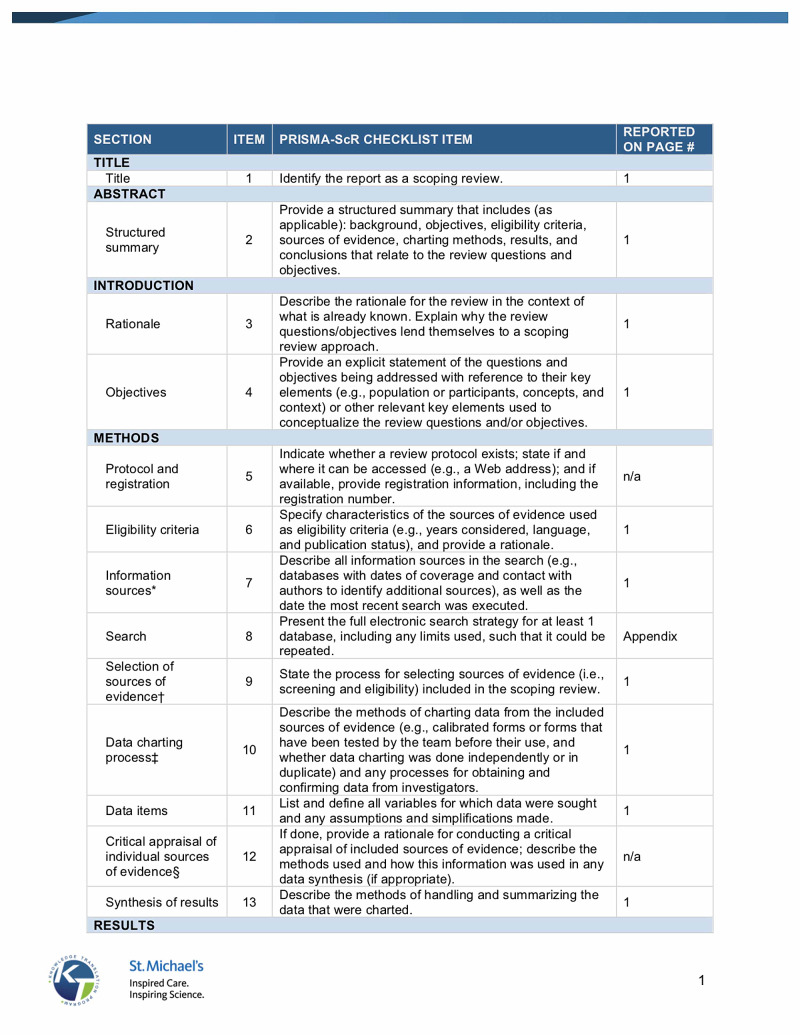
Preferred Reporting Items for Systematic reviews and Meta-Analyses extension for Scoping Reviews (PRISMA-ScR) Checklist Note – As the submission appears as a single web-page, the ‘Reported on Page #’ is 1 for most answers.

## Appendices

Appendix Published literature search executed on October 22, 2019 and June 26, 2020.

Embase

'adrenalin'/exp OR epinephrine:ab,ti OR adrenaline:ab,ti OR adrenalin:ab,ti AND (repeat:ti OR 'repeat epinephrine':ab,ti OR dose:ab,ti OR dosage:ab,ti OR doses:ab,ti OR 'second injection':ab,ti OR 'next injection':ab,ti OR '2 injections':ab,ti OR 'two injections':ab,ti OR twinject:ab,ti OR 'additional injection':ab,ti OR 'additional injections':ab,ti OR 'repeated injection':ab,ti OR 'repeated injections':ab,ti OR 'repeat injection':ab,ti OR 'repeat injections':ab,ti OR multiple:ab,ti) AND ('anaphylaxis'/exp OR 'anaphylactic shock'/exp OR anaphylaxis:ab,ti OR anaphylactic:ab,ti OR 'severe allergic reaction':ab,ti OR 'severe allergic reactions':ab,ti) NOT ('animal'/exp NOT 'human'/exp) NOT ([editorial]/lim OR [letter]/lim OR 'case eport'/de) AND [embase]/lim

PubMed

((((((("multiple dose"[TI] or "multiple doses"[TI] or repeat[TI] or second dose[TI] or second doses[TI])AND epinephrine[TI])))) OR (((((((("Epinephrine"[Mesh] OR Epinephrine[TIAB] OR Adrenaline[TIAB] or adrenalin[TIAB]))) AND (("administration and dosage" [Subheading] OR "therapeutic use" [Subheading:NoExp] OR "repeat epinephrine"[TIAB] OR dose[TIAB] OR dosage[TIAB] or doses[TIAB] or "second injection"[TIAB] or "next injection"[TIAB] or "2 injections"[TIAB] or "two injections"[TIAB] or Twinject[TIAB] or "additional injection"[TIAB] or "additional injections"[TIAB] OR "repeated injection"[TIAB] or "repeated injections"[TIAB] or "repeat injection"[TIAB] or "repeat injections"[TIAB] or multiple[TIAB])))) AND ((("Anaphylaxis"[Mesh] OR Anaphylaxis[TIAB] or anaphylactic[TIAB] or "severe allergic reaction"[TIAB] or "severe allergic reactions"[TIAB]) AND ("therapy" [Subheading:NoExp] OR "drug therapy" [Subheading] OR "prevention and control" [Subheading]))))))))NOT ((animals[mh] NOT humans[mh]) NOT ("letter"[pt] OR "comment"[pt] OR "editorial"[pt] or Case Reports[ptyp] ))

**Table 2:** Grey literature search strategy and results Google search conducted on November 18, 2019 and again on June 26, 2020.

#	Search	# results	# results screened	# new potentially relevant records	Total # records	# Included after review
1	anaphylaxis AND repeat epinephrine	~ 385,000	100	14	14	0
2	anaphylaxis AND repeat adrenaline	~ 137,000	100	3	17	0
3	anaphylaxis AND second dose epinephrine	~ 393,000	100	0	17	0
4	anaphylaxis AND second dose adrenaline	~ 277,000	100	0	17	0
5	anaphylaxis AND multiple doses epinephrine	~ 330,000	100	1	18	0
6	anaphylaxis AND multiple doses adrenaline	~ 273,000	100	1	19	0
